# On the regional distribution of cerebral microvascular ‘raspberries’ and their association with cerebral atherosclerosis and acute circulatory failure

**DOI:** 10.1016/j.cccb.2023.100157

**Published:** 2023-01-07

**Authors:** Henric Ek Olofsson, Mattias Haglund, Elisabet Englund

**Affiliations:** Department of Clinical Sciences Lund, Division of Pathology, Lund University, Sölvegatan 25B, Lund 22185, Sweden

**Keywords:** Cerebrovascular disease, Cerebral ischemia, Arteriosclerosis, C-ASCL, cerebral atherosclerosis, ACF, acute circulatory failure

## Abstract

**Introduction:**

In this follow-up study, cerebral microvascular formations termed ‘raspberries’ were quantified according to cerebral atherosclerosis (C-ASCL) and acute circulatory failure (ACF). We also examined the regional distribution of raspberries throughout the brain.

**Materials and methods:**

The study population consisted of adult individuals who had undergone a diagnostic neuropathological autopsy. Groups were formed to examine the association between raspberries, C-ASCL and ACF (control group, C-ASCL group, C-ASCL+ACF group [*n* = 47 per group] and a combined C-ASCL-tot group [*n* = 94]). To examine the regional distribution, additional groups were formed based on previously known raspberry densities of the frontal cortex (high-, medium- and low-density group [*n* = 6 per group]). Raspberries were quantified on scanned haematoxylin-eosin-stained sections.

**Results:**

Cortical raspberry density did not differ at a statistically significant level between the control group, the C-ASCL group and the C-ASCL+ACF group (*P* = 0.10) but did so between the control group and the C-ASCL-tot group (*P* = 0.033). The total raspberry density of the high-, medium- and low-density groups differed at a statistically significant level (*P* = 0.005), which remained in group-to-group comparisons of the high- and medium-density groups (*P* = 0.015) and the high- and low-density groups (*P* = 0.002). Raspberries were rare in cerebral white matter and in the cerebellum.

**Conclusion:**

An association between raspberry density and C-ASCL is supported but is weaker than previously indicated. An association with ACF is not indicated. The raspberry density of the frontal cortex provides an approximation of the brain's total raspberry density.

## Higlights

• A ‘raspberry’ is a microvascular formation that has been found in neuropathological autopsies.
• Raspberries were quantified according to cerebral atherosclerosis and acute circulatory failure.
• The regional distribution of raspberries throughout the brain was examined.
• There is a weak association between raspberries and cerebral atherosclerosis.
• Raspberries are rare in cerebral white matter and in cerebellum.

## 1 Introduction

**Fig. 1:**
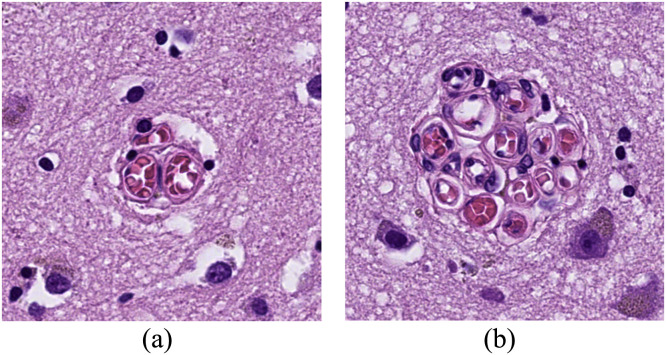
Two raspberries, measuring 30 μm in diameter (A) and 70 μm in diameter (B).

In two previous histopathological studies, we have examined a cerebral microvascular formation that we currently term ‘raspberries’ (Fig. 1) [1,2]. When viewed under a bright-field microscope, a raspberry consists of ≥ 3 adjacent vascular lumen, likely arterioles since the vessel walls generally stain positively for smooth muscle actin (unpublished data) [3]. The formations vary in size from 20 to 80 µm and occur in areas affected by pathology as well as areas that are free of adjoining reactive changes.

In our first study, we observed a higher raspberry frequency in the cerebral cortex of patients with major vascular cognitive impairment compared to Alzheimer's disease, frontotemporal lobar degeneration and control cases, as well as a higher frequency in the frontal cortex compared to the occipital cortex [1]. In our second study [2], we quantified cortical raspberry density according to clinical and pathological variables indicative of cerebral hypoperfusion and observed a higher raspberry density in subjects with atherosclerosis of the basal cerebral arteries (the basilar artery and the circle of Willis [4]), referred to here as cerebral atherosclerosis (C-ASCL). The raspberry density was especially high in a subgroup of patients who had suffered from acute circulatory failure (ACF) in addition to C-ASCL. In contrast, the raspberry density in subjects without C-ASCL was similar regardless of ACF status. Due to the low number of patients with the double exposure of C-ASCL+ACF, no statistical analysis was performed on this data. Patients with ACF included those with a history of shock, sepsis, cardiac arrest with return of spontaneous circulation, and cardiac surgery with perioperative extracorporeal membrane oxygenation.

Our previous study was exploratory and of limited size. The primary aim of the current study was to examine whether the raspberry density of the cerebral cortex is associated with C-ASCL and whether the association is affected by ACF in a large, independent and matched study sample. Our secondary aim was to further describe the regional distribution of raspberries throughout the brain and to evaluate the frontal cortex as a marker of the overall raspberry density of the brain.

## 2 Materials and methods

### 2.1 Study population

The study population of this retrospective study was drawn from adult patients who had undergone a clinical autopsy including a neuropathological examination at the Department of Pathology in Lund, Sweden.

For our primary aim, the patients were to be divided into three groups: absence of both C-ASCL and ACF (control group), presence of C-ASCL and absence of ACF (C-ASCL group) and presence of both C-ASCL and ACF (C-ASCL+ACF group). Prior to data collection, a sample size calculation was performed in Epitools (http://epitools.ausvet.com.au). The patients included in our latest study on this topic [2] were divided into three groups according to the same exposure variables as in the current work (control group, C-ASCL group and C-ASCL+ACF group). Based on the smallest observed mean difference between these groups (4.8 raspberries/cm²), the overall variance (53), a 98% confidence interval and 80% statistical power, this resulted in a required sample size of 47 patients per group. For some tests, the patients from the C-ASCL group and the C-ASCL+ACF group were combined, forming the C-ASCL-tot group (*n* = 94).

The groups were formed as follows: Starting in September 2021 and proceeding backwards, all consecutively received neuropathological autopsy cases were assessed for potential inclusion until each group consisted of ≥ 47 subjects. This resulted in an assessment of the period of January 1993–September 2021. Subjects were then matched across the three groups according to age at death (striving to achieve minimum age difference, resulting in +/– 3 years between matched subjects), sex (complete match achieved) and examined cortical region (complete match achieved; see 2.2. Raspberry quantification for details) until each group consisted of 47 matched individuals. To ensure independent study samples, cases included in our latest study on raspberries [2] were not included in the groups. However, cases included in our first study on raspberries [1] – a study that addressed other research questions – were considered eligible for inclusion. In total, 10 patients from our first study were included in the current work.

For our secondary aim, we examined a subgroup of the patients included in our previous study, in which the raspberry density of the frontal cortex had been quantified [2]. Based on this raspberry density, three groups of six patients each were formed, consisting of the individuals with the highest raspberry density (high-density group), the lowest raspberry density (low-density group) and those whose raspberry density was closest to the total mean (medium-density group).

### 2.2 Raspberry quantification

The raspberry quantification was performed as previously described [2]. In summary, 3-µm-thick tissue sections stained with haematoxylin-eosin that had been mounted on standard slides were scanned and viewed in Sectra IDS7 or Aperio ImageScope. The area of interest was digitally measured and examined for raspberries. A raspberry was defined as ≥ 3 transversally sectioned microvascular lumen in immediate proximity to one another and surrounded by a common space. Raspberry density was measured as raspberries/cm² and standardised through multiplication by the quotient of the individual brain weight divided by the sex-specific mean brain weight [5]. The raspberry quantification was done blinded to group affiliation. All quantification was based on tissue collected as part of the diagnostic work at this laboratory.

For the control group, the C-ASCL group and the C-ASCL+ACF group, one slide per patient was examined. When available, a slide representing the frontal cortex was selected for examination. When not available, slides from the parietal cortex, the occipital cortex or the hippocampus were used as substitutes.

For the high-density, medium-density and low-density groups, available tissue from the following regions was examined: parietal cortex, occipital cortex, hippocampus, basal ganglia (caudate nucleus and putamen), pons (posterior region), cerebellum (cortex and superficial white matter) and cerebral white matter (semioval centre). The previously documented raspberry density of the frontal cortex was noted but not included in the statistical analyses.

An exception from the method of raspberry quantification described above was made for slides representing cerebral white matter. These measurements were made on 6-µm-thick, bi-coronal tissue sections that were too large to scan and thus were viewed under a bright-field microscope. For each of these patients, an area of 16 microscopical fields at × 10 magnification was examined, corresponding to an area of 1 cm².

### 2.3 Cerebral atherosclerosis and acute circulatory failure

Cerebral atherosclerosis was defined as atherosclerosis of the basal cerebral arteries (the basilar artery and the circle of Willis). Data on C-ASCL was retrieved primarily from autopsy reports, where the occurrence of atherosclerotic plaques in the basal cerebral arteries is macroscopically assessed and routinely confirmed or denied. When not documented, tissue sections were examined microscopically by the authors to assess whether the atherosclerotic status of these arteries could be determined retrospectively. Cases where atherosclerotic status could not be determined were excluded from raspberry quantification. Autopsy reports were accessed through Sympathy (January 1993–March 2019) and LIMS RS (April 2019–November 2021), the digital systems in which all autopsies performed in Region Skane, Sweden, are documented.

The presence or absence of ACF was determined by assessing the patients’ autopsy referrals and medical records, and the definition of ACF was the same as in our previous study [2]. In summary, the status of this variable was determined based on clinical and biochemical findings established in consensus definitions of shock and sepsis [6,7]. Patients with cardiac arrest with return of spontaneous circulation were also considered to have had ACF, as were patients who had undergone cardiac surgery with perioperative extracorporeal membrane oxygenation [8,9]. As in our previous study, events occurring ≤ 2 weeks prior to death were excluded. Cases where ACF was suspected but could not be established based on the applied criteria were excluded from raspberry quantification. The medical records were accessed electronically through the digital system Melior, in which public hospital care in Region Skane, Sweden, is documented.

### 2.4 Statistics

The raspberry densities of the control group, the C-ASCL group, and the C-ASCL+ACF group were compared using non-parametric tests due to skewed data. Likewise, non-parametric tests were performed to compare the high-, medium- and low-density groups. When indicated, a Bonferroni correction was applied to account for multiple tests.

Potential associations between cortical raspberry density, age and brain weight were analysed with simple linear regression on all individuals of the control group, the C-ASCL group and the C-ASCL+ACF group. The potential association with brain weight was examined using unstandardised raspberry densities.

The statistical tests were performed in IBM SPSS Statistics Version 28. A P value of ≤ 0.05 was considered statistically significant.

### 2.5 Ethical approval

The study was approved by the Swedish Ethical Review Authorities, application number 2021-05753-02.

## 3 Results

As previously described, the control group, the C-ASCL group and the C-ASCL+ACF group had been matched for age at death, sex and examined brain region. The mean age of all three groups was 76 years (range 57–92 years), and 74% of the patients were male. A total of 85% of the examined tissue sections were from the frontal cortex, 4% from the hippocampus, 6% from the parietal cortex and 6% from the occipital cortex. The mean examined cortical area was 1.94 cm².

**Fig. 2:**
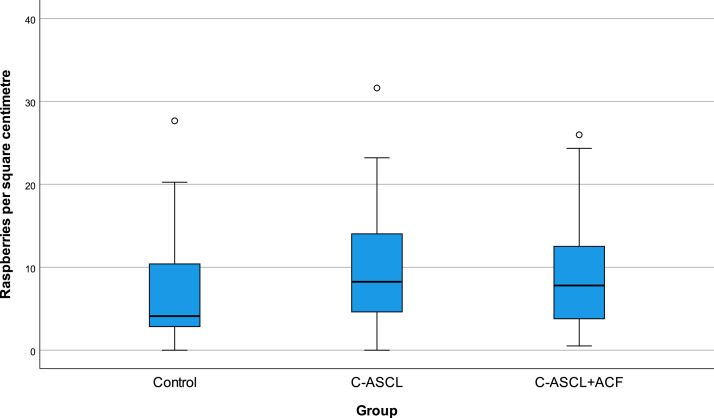
Raspberry density (raspberries/cm²) of the control group, the C-ASCL group and the C-ASCL+ACF group (*n* = 47 per group). C-ASCL = cerebral atherosclerosis. ACF = acute circulatory failure.

The mean cortical raspberry density was 7.2 (range 0–27.7) raspberries/cm² for the control group, 9.6 (range 0–31.6) for the C-ASCL group, 9.1 (range 0.5–26.0), for the C-ASCL+ACF group and 9.3 (range 0–31.6) for the C-ASCL-tot group. Raspberry density was positively skewed rather than normally distributed (Fig. 2), especially in the control group, resulting in larger differences in median than in mean. The median was 4.1 raspberries/cm² in the control group, 8.3 in the C-ASCL group, 7.8 in the C-ASCL+ACF group and 8.3 in the C-ASCL-tot group. It was noted that cases where the C-ASCL had been described as severe in the autopsy report (*n* = 10) had a higher raspberry density, with a median of 13.2 (range 0.9–23.2) raspberries/cm² compared to 8.2 (range 0–31.6) for the rest of the C-ASCL-tot group (*n* = 84).

The Kruskal–Wallis test performed on the control group, the C-ASCL group and the C-ASCL+ACF group was statistically inconclusive (*P* = 0.10). When comparing the control group to the C-ASCL-tot group, a statistically significant difference was observed (*P* = 0.033).

The cortical raspberry density exhibited a weak and statistically inconclusive association with age (*P* = 0.063, β = 0.12), and no association was observed between unstandardised raspberry density and brain weight (*P* = 0.18, β = 0.01).

Background data and regional distribution of raspberries for the high-, medium- and low-density groups is presented in Table 1. The mean examined area (frontal cortex excluded) was 9.9 cm². Total median raspberry density (frontal cortex excluded) was 7.3 (range 3.7–10.7) raspberries/cm² for the high-density group, 3.2 (range 1.1–4.3) for the medium-density group and 1.8 (range 1.6–2.9) for the low-density group. The Kruskal–Wallis test demonstrated statistically significant differences between the groups (*P* = 0.005); consequently, group-to-group comparisons were performed (Mann–Whitney, with a significance level of *P* = 0.05 corresponding to *P* = 0.017 after the Bonferroni correction). The differences remained statistically significant when comparing the high- and medium-density groups (*P* = 0.015) as well as the high- and low-density groups (*P* = 0.002). The difference between the medium- and low-density groups did not reach statistical significance (*P* = 0.24).

**Table 1:** Background data and regional distribution of raspberries for the high-, medium- and low-density groups. These groups were formed based on previously known raspberry densities of the frontal cortex, which are reported in the table* but not included in the total raspberry density. Raspberry density (raspberries/cm²) is presented as median and range.

	High-density group	Medium-density group	Low-density group
**Background data**			
Mean age at death	78 (range 67–89)	77 (range 68–90)	73 (range 66–88)
Female/Male	2/4	1/5	1/5
**Regional distribution of raspberries**			
Hippocampus	6.8 (2.0–11.1), *n* = 6	1.5 (0.0–4.6), *n* = 6	0.6 (0.0–5.1), *n* = 6
Parietal cortex	7.1 (2.1–9.6), *n* = 4	4.3 (0.0–13.1), *n* = 5	1.1 (0.7–2.9), *n* = 5
Occipital cortex	8.0 (5.2–10.5), *n* = 5	1.5 (0.0–3.5), *n* = 6	0.8 (0.0–2.2), *n* = 5
Basal ganglia	11.8 (4.8–29.9), *n* = 6	4.1 (1.9–12.7), *n* = 5	4.8 (3.3–6.9), *n* = 6
Pons	9.2 (2.2–16.5), *n* = 6	3.3 (1.6–6.1), *n* = 6	1.8 (0.9–2.0), *n* = 5
Cerebellum	0.9 (0.0–1.8), *n* = 6	0.0 (0.0–0.0), *n* = 6	0.0 (0.0–0.0), *n* = 6
White matter	0.0 (0.0–2.0), *n* = 5	0.0 (0.0–0.9), *n* = 5	0.0 (0.0–1.1), *n* = 6
Total	7.3 (3.7–10.7), *n* = 6	3.2 (1.1–4.3), *n* = 6	1.8 (1.6–2.9), *n* = 6
Frontal cortex*	22.0 (20.9–35.7), *n* = 6	7.3 (7.1–7.6), *n* = 6	1.9 (1.4–2.2), *n* = 6

## 4 Discussion

### 4.1 Raspberries, cerebral atherosclerosis and acute circulatory failure

The main part of this study was a follow-up to a smaller study that demonstrated higher raspberry density in patients with C-ASCL compared to control cases (statistically significant) and even higher in the C-ASCL+ACF subgroup (not analysed due to the small sample size) [2]. Examining these potential associations in a larger and independent study sample did not demonstrate statistically significant differences between the C-ASCL group, the C-ASCL+ACF group and the control group. Due to the highly similar raspberry density of the C-ASCL group and the C-ASCL+ACF group, ACF as defined in this study is concluded not to be associated with raspberry density.

Since studies with low power have a risk of overestimating effect sizes [10] and thereby underestimating sample size, we also compared the control group to the larger C-ASCL-tot group. In this comparison, a statistically significant difference was indeed observed. Consequently, we conclude that there is an association between raspberry density and C-ASCL but that it is weaker than indicated by our previous study [2].

### 4.2 How is the association between raspberries and cerebral atherosclerosis to be interpreted?

We have previously hypothesised that raspberries are a sign of adult cerebral angiogenesis induced by an ischaemic stimulus and have suggested mild hypoperfusion as such a stimulus due to the frequent absence of damage to the surrounding parenchyma. Cerebral hypoperfusion can be caused by hypotension, the effects of which can be reduced through cerebral autoregulation. We have speculated in causality between C-ASCL and raspberry formation, such that atherosclerosis would increase the susceptibility to cerebral hypoperfusion during episodes of hypotension due to impaired autoregulation. Part of cerebral autoregulation has indeed been indicated to take place at the level of the basal cerebral arteries [11], and atherosclerosis can contribute to atrophy of vascular smooth muscle cells [12], the primary mediators of cerebral autoregulation [13]. The inconclusive finding of a higher raspberry density in a subgroup of patients with severe C-ASCL is interesting in this regard and it should be considered when conceiving future studies. However, our results also demonstrate that raspberries can be numerous in the absence of C-ASCL and that not all individuals with C-ASCL have high raspberry density, arguing against C-ASCL as the predominant cause of raspberry formation. The results are limited by the dependence on retrospectively collected data from autopsy protocols for the determination of atherosclerotic status.

Another hypothesis is that cortical raspberries and C-ASCL are both signs of cerebrovascular disease that arise independently of one another. That raspberries are a sign of cerebrovascular disease would be in line with our previous finding of an association between raspberries and major vascular cognitive impairment [1]. According to this hypothesis, one might expect an association between raspberry density and other markers, risk factors or consequences of cerebrovascular disease as well. However, a more recent study of ours failed to show an association between raspberry density and clinically reported hypertension and diabetes mellitus [2]. Identifying such associations might be challenging with the current retrospective, observational design due to the reliance on data reported for clinical rather than scientific purposes. Obtaining reliable data on risk factors such as diabetes mellitus and hypertension might be further complicated since they might exert their major influence decades prior to death [14].

Our hypothesis that raspberries are induced by cerebral ischaemia needs to be addressed in future studies, as does the potential clinical implications of raspberries. The focus of our next study will be to analyse raspberry-dense tissue samples with immunohistochemical markers, including markers of angiogenesis and parenchymal damage.

### 4.3 Regional distribution of raspberries and their relation to other vascular formations

In this context, some microvascular formations similar to raspberries will be mentioned [15], [16], [17], [18], [19], [20], [21]. While some of these formations share common aspects with raspberries – and possibly a degree of overlap due to the broad definition of raspberries – there are also some distinctions. Raspberries were easily found in the neocortex, the hippocampus, the basal ganglia and the brainstem but only rarely in cerebral white matter and in the cerebellum. This resembles the distribution of similar arteriolar formations referred to as ‘vascular bundles’ and ‘wickerworks’ [15] but separates raspberries from arteriolar ‘tortuosity’, which primarily occurs in white matter [19], [20], [21]. Like raspberries, vascular bundles and wickerworks exhibited a weak association with cerebral atherosclerosis [16]. Unlike studies on the abovementioned vascular alterations, we have found only a weakly positive and statistically inconclusive association between raspberries and age in this and previous works. However, we have examined only individuals of middle age and older. Additionally, we found no sign of an association between raspberry density and brain weight, arguing against the notion that raspberry density would be affected by brain atrophy.

A notably high raspberry density was observed in the basal ganglia (caudate nucleus and putamen, descriptive data). As structures frequently affected by small vessel disease [22,23] and believed to be particularly susceptible to hypertension due to the anatomy of their blood supply [13], the basal ganglia could be an area of interest for examinations of potential correlations between raspberries and small vessel disease.

The total raspberry density (frontal cortex excluded) differed at a statistically significant level between the groups. This statistical significance was lost when comparing the medium- and low-density groups but remained intact in the other group-to-group comparisons, and the region-specific differences between the groups resembled those observed in the frontal cortex. The results indicate that the raspberry density of the frontal cortex provides an approximation of the brain's total raspberry load on a group level. While the patients were included based on the raspberry density of the frontal cortex (high, medium and low), the fact that this region had the highest to second highest raspberry density in all the groups (descriptive data) indicates that the frontal cortex is raspberry dense relative to other regions.

### 4.4 Strengths and limitations

This study is a pertinent follow-up to our previous study, such that the potential associations between raspberries, C-ASCL and ACF have been examined in a larger, independent and matched study sample. Moreover, the regional distribution of raspberries throughout the brain has not been previously described.

In addition to the problems of retrospective observational design discussed above, it is a limitation that we use markers indicative of ACF rather than direct measures thereof. Originally, we considered a study design that would focus on survivors of cardiac arrest, as an easy-to-define marker of global hypoperfusion. However, the prevalence of this condition in individuals referred to autopsy was too low to provide the desired number of patients. Furthermore, the proportion of women in this study was small, since more men than women undergo autopsy. Finally, the application of non-parametric rather than parametric testing reduced the statistical power of the study.

## 5 Conclusion

Our results confirm an association between cortical raspberry density and C-ASCL, but this association is weaker than was indicated by our previous study. An association between raspberry density and ACF is not indicated. Raspberries occur in several parts of the brain but are rare in cerebral white matter and in the cerebellum. The raspberry density of the frontal cortex provides an approximation of the brain's total raspberry density on a group level.

## Funding

This work was supported by the Trolle-Wachtmeister Foundation for Medical Research and Region Skane.

## CRediT authorship contribution statement

**Henric Ek Olofsson:** Data curation, Writing – original draft, Conceptualization, Writing – review & editing. **Mattias Haglund:** Conceptualization, Writing – review & editing. **Elisabet Englund:** Conceptualization, Writing – review & editing.
